# Participatory Action Research-Dadirri-Ganma, using Yarning: methodology co-design with Aboriginal community members

**DOI:** 10.1186/s12939-021-01493-4

**Published:** 2021-07-12

**Authors:** Hepsibah Sharmil, Janet Kelly, Margaret Bowden, Cherrie Galletly, Imelda Cairney, Coral Wilson, Lisa Hahn, Dennis Liu, Paul Elliot, Joanne Else, Trevor Warrior, Trevor Wanganeen, Robert Taylor, Frank Wanganeen, Jodus Madrid, Lisa Warner, Mandy Brown, Charlotte de Crespigny

**Affiliations:** 1 Chettinad College of Nursing (CCN), Chettinad Academy of Research and Education (CARE), Chettinad Health City, Rajiv Gandhi Salai, OMR, 603103, Kelambakkam, Tamil Nadu Chennai, India; 2 Dr. M.G.R Educational and Research Institute, Maduravoil, Chennai India; 3 Adelaide Nursing School, The University of Adelaide, South Australia Adelaide, Australia; 4 Em Bee’s Editing. Retired, South Australia Adelaide, Australia; 5 Discipline of Psychiatry, School of Medicine, The University of Adelaide, South Australia Adelaide, Australia; 6 Ramsay Health Care (SA) Mental Health Services, The Adelaide Clinic, South Australia Adelaide, Australia; 7 Northern Adelaide Local Health Network, South Australia Adelaide, Australia; 8 Ramsay Health Care (SA) Mental Health, South Australia Adelaide, Australia; 9 Central Adelaide Local Health Network, Older Persons Mental Health Services, South Australia Adelaide, Australia; 10 Co-researcher, Cultural Advisor, Adelaide, South Australia Australia; 11 Aboriginal Working Party (AWP) Members, Aboriginal Comorbidity Action in the North (CAN) Project, The University of Adelaide, South Australia Australia, Australia

**Keywords:** Participatory action research (PAR), Ganma, Dadirri, Yarning, Aboriginal knowledge, Aboriginal, Methodology, Indigenous, Comorbidity, Substance abuse, Alcohol, Mental health

## Abstract

**Background:**

Appropriate choice of research design is essential to rightly understand the research problem and derive optimal solutions. The Comorbidity Action in the North project sought to better meet the needs of local people affected by drug, alcohol and mental health comorbidity. The aim of the study focused on the needs of Aboriginal peoples and on developing a truly representative research process. A methodology evolved that best suited working with members of a marginalised Aboriginal community. This paper discusses the process of co-design of a Western methodology (participatory action research) in conjunction with the Indigenous methodologies Dadirri and Ganma. This co-design enabled an international PhD student to work respectfully with Aboriginal community members and Elders, health professionals and consumers, and non-Indigenous service providers in a drug and alcohol and mental health comorbidity project in Adelaide, South Australia.

**Methods:**

The PhD student, Aboriginal Elder mentor, Aboriginal Working Party, and supervisors (the research team) sought to co-design a methodology and applied it to address the following challenges: the PhD student was an international student with no existing relationship with local Aboriginal community members; many Aboriginal people deeply distrust Western research due to past poor practices and a lack of implementation of findings into practice; Aboriginal people often remain unheard, unacknowledged and unrecognised in research projects; drug and alcohol and mental health comorbidity experiences are often distressing for Aboriginal community members and their families; attempts to access comorbidity care often result in limited or no access; and Aboriginal community members experience acts of racism and discrimination as health professionals and consumers of health and support services. The research team considered deeply how knowledge is shared, interpreted, owned and controlled, by whom and how, within research, co-morbidity care and community settings. The PhD student was supported to co-design a methodology that was equitable, democratic, liberating and life-enhancing, with real potential to develop feasible solutions.

**Results:**

The resulting combined Participatory Action Research (PAR)-Dadirri-Ganma methodology sought to create a bridge across Western and Aboriginal knowledges, understanding and experiences. Foundation pillars of this bridge were mentoring of the PhD student by senior Elders, who explained and demonstrated the critical importance of Yarning (consulting) and Indigenous methodologies of Dadirri (deep listening) and Ganma (two-way knowledge sharing), and discussions among all involved about the principles of Western PAR.

**Conclusions:**

Concepts within this paper are shared from the perspective of the PhD student with the permission and support of local Elders and Working Group members. The intention is to share what was learned for the benefit of other students, research projects and community members who are beginning a similar journey.

## Background

Many Indigenous peoples, within Australia and worldwide, often view Western research as untrustworthy and unwelcome [1]. This is due to research being conducted ‘on’ Aboriginal people as part of colonising practices, solely for the benefit of researchers, while Indigenous priorities, benefits and research approaches are ignored [2–5].

This paper discusses the process of co-designing a Participatory Action Research (PAR) methodology that combined Western and Indigenous understandings of what constitutes good collaborative research in relation to improving local health service delivery. The South Australian multi-phase PAR ‘Stopping the Run Around’: Comorbidity Action in the North (CAN) project sought to make recommendations for improvements to local mental health and alcohol and other drugs (MH-AOD) comorbidity services for people aged 12 years and over. When no Aboriginal or local non-Aboriginal researcher applied to conduct the Aboriginal arm of the project, the main author (HS), at the time an international PhD student, was engaged to undertake the work. The innovative methodology was developed to enable her to work respectfully and effectively with Aboriginal community members in the northern suburbs of Adelaide [2].

When considering the best approach to choosing or designing a methodology, several challenges needed to be considered. The main author was an international student with no existing relationship with local Aboriginal community members or pre-existing Aboriginal cultural knowledge. It was imperative that the Aboriginal community involved in the research did not see her as just another non-Indigenous researcher imposing another project on them from a purely Western-oriented, colonising research approach [5]. She realised the need for the Aboriginal community members to have a sense of ownership of the research because it was about a problem affecting them, their experiences and seeking solutions they thought would work. Such an approach has been well documented in an extensive review of the roles non-Indigenous and Indigenous researchers play in Australian health research, as have capacity-building strategies for Indigenous researchers [6]. Another consideration was the deep distrust of Western research held by many Aboriginal people [6, 7], resulting from repeated experiences of being unheard, unacknowledged and unrecognised in research and health services planning and reform [8]. There was also frustration with the lack of implementation of research findings into practice; a global issue in health service delivery [9]. In addition, the subject matter of this research project was drug and alcohol and mental health comorbidity experience, often distressing for Aboriginal community members and their families [10]. Attempts to access comorbidity care had all too often resulted in limited, culturally inappropriate care or no access [10]. Also, in contemporary Australia, Aboriginal community members experience acts of racism and discrimination as health professionals and as consumers of health and support services [11].

### Gaining cultural knowledge from an Aboriginal mentor and an Aboriginal Working Party

The CAN project research team included a respected Adelaide Plains Kaurna Aboriginal Elder Aunty Coral Wilson (ACW)^2^ who had worked extensively in the area of research, drug and alcohol, and mental health. The international student (HS) travelled from India following her passion to learn best nursing practice and to contribute to health care research. HS worked as a Registered Nurse in the Emergency Department of a hospital in the northern suburbs of Adelaide. She applied for doctoral studies to learn how to undertake research from expert researchers. She was offered the Aboriginal CAN project and informed that she could only undertake it if ACW and local Aboriginal community members accepted her. This project had not been taken up by any candidate for over two years. The principal supervisor introduced HS to the research team, who then invited her to undertake the project. ACW became HS’s mentor, explaining what was important from an Aboriginal perspective and what was culturally appropriate. As mentor, ACW facilitated HS by introducing her to community members and key stakeholders. Six months later an Aboriginal Working Party (AWP) was formed, consisting of ten local Aboriginal people with MH-AOD experience, who were interested in becoming co-researchers in the project. This group represented different genders and ages (AWP members 18 years and over, representing people of all ages), and diverse Aboriginal family and cultural groups. Major AWP meetings were held bi-monthly, with phone conversations, emails and face-to-face meetings in between. This flexible partnership enabled information sharing, problem solving and networking. HS had discussions with group members about what the research focus should be, how the research should be conducted, who else from the community should be involved and how they might engage with the project. The research approach was contextualised within the everyday needs and stressful events that affect families when caring for their MH-AOD dependent family members. This was communicated to the wider Aboriginal community with the assistance and guidance of mentor ACW and the AWP.

#### Ethics

Ethical approval was gained from an Aboriginal-specific ethics committee, the government health department, and non-government MH and AOD organisations. This was a long and complex 18-month process. It involved multiple discussions with key stakeholders, gaining letters of support from multiple community members, groups, and MH and AOD services, and meeting national and state guidelines for conducting Aboriginal Health Research, including the National Health and Medical Research Council (NHMRC) of Australia’s ethical principles and values that guide research involving Australian Aboriginal people [12], which promotes actively involving participants in all phases of the research and legitimising their ‘lived experiences’. The Aboriginal Health Research Ethics Committee of South Australia also has specific requirements and advised HS to collaborate and consult with the mentor (ACW) as co-researcher and with the AWP as an Aboriginal Reference group in each phase of the research process. When ACW was unavailable, another AWP member or local Aboriginal community member accompanied HS to meetings. This was openly discussed and agreed by all involved. Non-Indigenous researchers de Crespigny, Emden et al. [13] described a *Partnership model for ethical Indigenous research* that provided a culturally-safe, holistic, ethically-sound Aboriginal research approach with four key features for creating collaborative engagement with Aboriginal people; ‘Respect’, ‘Collaboration’, ‘Active Participation’ and ‘Meeting Needs’; concepts linked closely to the NHMRC ethical guidelines for Aboriginal research [12] and the Australian Institute of Aboriginal and Torres Strait Islander Studies (AIATSIS) ‘Code of Ethics’ [14].

## Methods

### Participatory action research

**Fig. 1:**
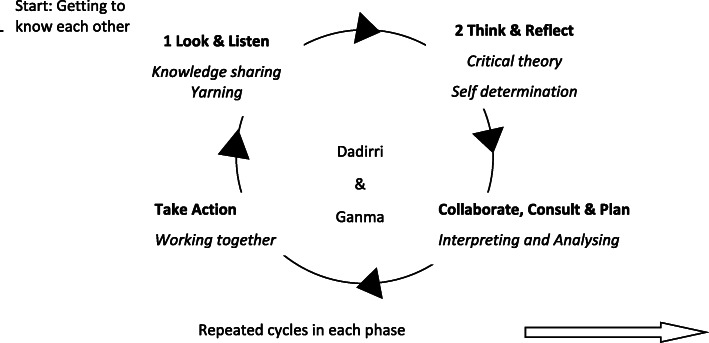
PAR inquiry cycle incorporating DADIRRI and GANMA

**Fig. 2:**
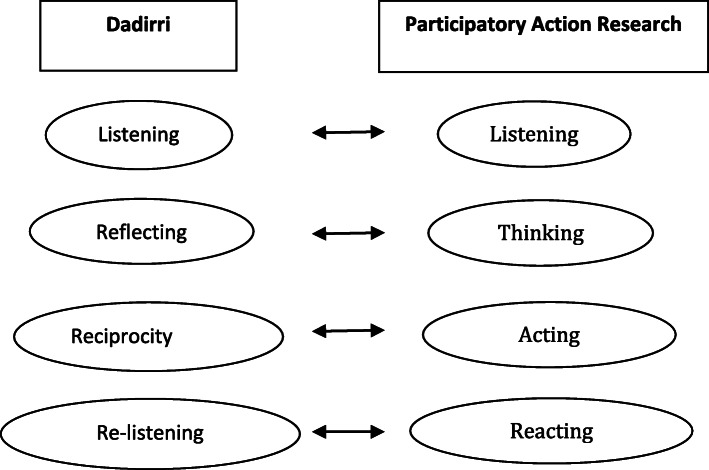
Components of Dadirri discovered by Hepsibah. S, and the PAR cycle ([2], p. 376)

While the wider CAN project had determined that a PAR approach (cycles of Look and Listen; Think and Reflect; Collaborate, Consult and Plan; and Take Action) was required for each phase [15], the approach and details for the Aboriginal arm of the project were still to be determined. As with the wider project, PAR was selected as the preferred collaborative approach but with the addition of *knowledge sharing using Yarning, Dadirri and Ganma* in the Look and Listen phase, and *Dadirri and Ganma* particularly in the Collaborate, Consult and Plan phase as well as throughout the entire project (see Table 1; Figs. 1 and 2 for full details of PAR with Yarning, Dadirri and Ganma methodology and methods) because, from the participants’ perspective, it enabled deeper understanding of the research problem in order to find appropriate and responsive action-oriented solutions [16].

**Table 1:** PAR methodologies and methods over 4 phases

Methodology	Methods
***Phase 1***: ***Look and listen*** *–knowledge sharing using Yarning, Dadirri and Ganma*, listening carefully to a diverse range of participants. The participants consist of three groups who were all involved in comorbidity care in the region; Group A- Participants from the local Aboriginal community who would act as consumer advocates, Group B – clinicians and workers from local MH and AOD services (government and non-government), and Group C – Workers from local support services (including emergency departments, ambulance and Aboriginal Health Workers, service coordinators, managers).	Data collection Meetings with - Aboriginal community members and groups, formal and informal discussions, building relationships - local MH-AOD clinicians and workers - support service staff a three-step process by HS and ACW with all participants 1. visit and introduce the project 2. in-depth conversation style interview or focus group 3. checking back that the manuscript was correct (member checking)
***Phase 2: Think and reflect***; stepping back and reflecting on the shared knowledge using critical theory from both a Western and Indigenous understanding, with consideration of colonisation impacts. Deep consideration regarding access to culturally-appropriate MH-AOD services, and research questions such as: How are MH-AOD services structured? Does this benefit Aboriginal consumers? How easy or difficult is it for Aboriginal consumers to get access to these services?	Data analysis – a collaborative process between HS and ACW - Systematically organised contextual thematic analysis - Interactive coding and categorising as themes Identify - Existing gaps in care, from multiple perspectives - Strategies and services that are meeting Aboriginal comorbidity needs. - Suggestions for improvement
***Phase 3: Collaborate, consult and plan*** *using Dadirri and Ganma*; interpreting and analysing the data together, and including the diverse knowledge, ideas and concepts shared. Concepts of mutual partnership ensured that the needs, perceptions and opinions of each person were considered, and no one person ruled over another, and no one person’s knowledge was considered more important than another’s. This needs to be a carefully negotiated approach that respects the role of senior Elders and Aboriginal consumers, yet also gives space for a range of voices and opinions to be heard. Rather than a step-by-step process following a set systematic (Western) formula, this process is based on mutual partnerships and respectful inclusion, discussion, disagreement and consensus making. This phase uses living knowledge to inform change, inviting participants and key stakeholders to work together to create an advanced, deeper level understanding and a practicable outcome.	Collaborative process led by HS and ACW Emerging themes discussed with: - Aboriginal Working Party - Aboriginal consumer, MH-AOD & support service participants - Wider CAN research team. Confirm findings with participants in a CAN Aboriginal workshop involving all participants and the wider Aboriginal community Discuss findings with participants and community members in an open public forum for them to confirm, refute or agree upon, in order to come up with the most appropriate solutions to best meet the Aboriginal community’s MH-AOD needs.
***Phase 4: Take action***; a reflective cycle of consultation and action, which is repeated until a solution is reached [17]. This phase involves carrying out the agreed plan of action in a collaborative, systematic, logical and appropriate way, and critically reflecting on each step. The process of “trustworthy action” arises through participation-mutual consultation, collaboration and collective reflection towards the collaboratively-agreed goal [16].	Community report of findings. Agreed recommendations for Aboriginal MH-AOD improvements and implementation in the local region

As HS began to explore possible approaches to the research, she became aware of the need to incorporate the diverse voices of local Aboriginal community members. Through conversation and reading, and cross checking with local Aboriginal people and non-Aboriginal people working closely with the Aboriginal community, HS discovered that Aboriginal people had a more holistic view of life and connectedness within their communities, with nature, and with their country or land [18, 19]. She realised that she would need to go beyond Western interpretations of PAR in order to respond to the advice of her mentor and the AWP, and Indigenous Research ethics guidelines. As she learned more, HS also reflected on her own cultural, educational and professional background, asking herself how these may influence her interpretations of what her Aboriginal research partners were teaching her. HS found the research process difficult initially but gradually came to understand the Aboriginal researchers’ ways of thinking, communicating and doing. ACW held HS’s hand throughout the entire process.

#### Yarning (consultation)

HS sought to create a research approach underpinned by Aboriginal values and culture that was reciprocal and involved transparent knowledge sharing [19]. It was essential to *truly* consult the local Aboriginal people about their needs and priorities, then present their opinions in their own voices, without the filter of her or other researchers’ assumptions [4]. The research process began with consultations in the form of “Yarning”, which involves a free-flowing, uninhibited conversation and deep listening in an environment in which (the intention was) all participants felt safe and respected [20]. Yarning promoted active participation and interaction, and strengthened partnerships, communication, responsibility and accountability; it created a space where the whole team and community members were able to face ongoing challenges together. Using Yarning principles made it possible to employ respectful and collaborative approaches to data collection, interpretation and categorisation of findings, enabling the best opportunity for potential application of realistically implementable, on-the-ground solutions [21].

#### ‘Dadirri’ (deep listening): Establishing trust

The next step involved gaining an understanding of the importance and process of deep listening. ‘Dadirri’ [22] is an Aboriginal word meaning inner, deep, quiet listening and a profound awareness of the “deep spring of sentience that comes from within”; it brings peace, understanding and increased awareness [23]. Dadirri is a concept that the Ngangikurungkurr (river people) from Daly River in the Northern Territory of Australia have chosen to share. Dr Ungunmerr-Baumann, an Elder of Daly River who was also the Principal of Daly River School, explains that Aboriginal people have endured learning the Western way and listening to what Western people say for many years, and while much of this was acceptable, some was obligatory; Aboriginal people were forced to listen. She said, “We still wait for fellow Australians to take time to know Aboriginal people and to be still and to listen to us” [24]. She insisted that listening and learning must go both ways; Aboriginal and Western knowledge must come together without one ruling the other.

In simpler terms, Dadirri means patient listening with understanding to enhance real communication, which is the heart of conversation. Dadirri encourages transparency about who we are and what we hope to achieve, and in research, what benefit research brings and for whom [25]. Dadirri recognises that individuals who are more ‘comfortable’ with each other build mutual trust, and exchange information more effectively than individuals who have less contact or are less at ease. Thus, Dadirri enables a trusting and respectful relationship to be built and maintained.

Reflecting deeply on Dr Ungunmerr-Baumann’s words, HS acknowledged that it was important to recognise and respect Aboriginal knowledge and incorporate it into the development and enacting of the methodology. HS realised that the fundamental elements of Dadirri, of mutually respectful interpersonal and social interactions, were important due to the past and ongoing impact of colonisation; from initial invasion, to Stolen Generations when Aboriginal children were forcibly removed from their families [23], to ongoing marginalisation and racism. HS discovered the hidden fact that not a single Aboriginal family she spoke to was untouched by the impact of these events, with resulting mental health and alcohol and drug implications. All shared these stories. HS felt humbled by this insight into the historical context behind the research project. Knowing this history and current impact was essential to understanding unique spiritual and cultural attributes, and the challenges many Aboriginal people experienced. These challenges, however, remain misunderstood and unrecognised by the majority of non-Aboriginal people [26]. HS acknowledged that to do no harm, and to avoid colonising assumptions and trends within this research project, it was important for her first to listen deeply and attentively.

#### ‘Ganma’: knowledge sharing

The Yolgnu people from Arnhem Land in the Northern Territory of Australia describe Ganma as respectful two-way sharing of cultural knowledge and interaction between Aboriginal and non-Aboriginal people [27]. Ganma refers to both a naturally occurring phenomenon involving two river systems on Yolgnu lands, and a way to improve relationships between Aboriginal people and non-Aboriginal people [28]. ‘Aboriginal knowledge’ represents water from the river (fresh water), and ‘Western knowledge’ (non-Aboriginal knowledge) represents water from the sea (salt water). When these waters run together at an interface they mix with each other to form a foam that is the creation of new knowledge generated from the interaction and collaboration of Aboriginal and Western knowledge [29]. By sharing their cultural understanding of Ganma, Aboriginal people have also shared how Aboriginal and Western peoples and knowledges can collaborate while maintaining their separate identities.

Ganma describes each person as being mindful of the other’s individual and combined experiences, and their contribution to the collaboration [27]. It provides the pathway for connecting people and bringing them to actively work together to create new knowledge that is not claimed as ‘mine’ or ‘yours’, but as ‘ours’ [30]. The process of knowledge-sharing and interaction has memory. Forgetting people’s history can lead to losing one’s identity [31]. The foam retains individual particles of both fresh water and salt water, which continue to carry their own identities and memory. The Yolgnu people explain that if the foam (knowledge) is cupped roughly in the hands, it evaporates; it must be held gently to reveal its true nature. It is also necessary to be quiet and patient, and to listen deeply to hear the foam’s soft sound [31]. In this way, Ganma is closely linked to Dadirri.

HS reflected that similarly, ACW and members of the local AWP had explained to her that for people to understand and work with Aboriginal ways of living and culture, they need to ‘work with sheer good heart (understanding), mind (attitude) and hands (skill) to render sharing hands to walk together’ [32], respecting the integrity of both Aboriginal and non-Aboriginal cultures [30]. When HS took these concepts back to ACW and AWP members for discussion, all identified that linking PAR with Ganma was justified and respectful. Although this knowledge sharing concept originated on the other side of Australia, the key elements of Ganma resonated with their own cultural understandings and philosophy. There was recognition that combining Ganma and PAR would enable a cross-cultural community development approach that recognised the importance of local Aboriginal people identifying and defining the problem requiring research; it would also prevent external researchers from working in isolation from the community [13, 33].

### PAR-Ganma research approach

PAR-Ganma enabled deeper exploration of the complex situations that were occurring for Aboriginal peoples in relation to MH and AOD morbidities. This provided potential for the health and wellbeing of the local community to improve through involvement in research; if their stories were heard, and their knowledge was respectfully incorporated, the results should inform culturally safe and responsive improvements in service provision. The suburbs where this research was situated are recognised as having significant socioeconomic disadvantage. Combining PAR with Ganma could help to describe the landscape of social and economic arrangements, as well as cultural implications, to identify why ‘health for all’ is not always possible for all community groups within capitalist societies [26]. The Ganma-PAR process also had the potential to bring together the knowledge and experiences of consumers and MH-AOD comorbidity service providers, enabling a more balanced understanding of the realities of health issues and health service responses. Potentially this process could also assist service providers to critically analyse their service provision and take appropriate action to improve their services in response to community information and feedback.

### Including key stakeholders

Collectively HS, ACW, the AWP members and the wider CAN research team also recognised the importance of involving other key stakeholders during the development of the research project to ensure engagement, support and participation [32]. Making meaningful changes in MH-AOD comorbidity service delivery required inclusion of a diverse range of knowledges – those of government and non-government service providers, clinicians, managers, coordinators and support services – and their roles. Working relationships needed to be built and maintained. HS began meeting with different people and groups providing co-morbidity services and support services, building relationships and inviting suggestions for the project. This process of inclusion of services began with the ethics approval process and continued through all phases of the research.

### Underpinning critical theory

Critical theory was identified as the most appropriate approach for this action-oriented project because critical theory encourages the questioning of power, socio-political and economic ideologies [33]. In particular, Habermas, a second-generation critical theorist, introduces the idea of emancipation through mutual understanding, appropriate communication and critical reflection [33]. Crotty suggests that critical research could uncover hidden domination and oppression, and enable exposure and analysis of power systems, thus contributing to liberation through change [34]. Freire [35] argues that marginalised people’s wisdom and knowledge is the best resource for achieving realistic solutions to the issues they encounter in everyday life. This resonates with processes of self-determination that give voice to Aboriginal people, rather than having others talking on their behalf [36]. Critical theory promotes liberation and identifies and challenges power structures [37]; it ‘works towards social change and trying to improve current reality through understanding’ [19]. As such, HS recognised that using critical theory could encourage consumers and service providers to look deeply into the MH-AOD service provision system to analyse consumer utilisation, satisfaction and benefit; it had the potential to support Aboriginal people’s self-empowered action for transformative change in their health status. Using critical theory as a basis, the inclusion of Aboriginal members to share their wisdom and knowledge about matters of concern for the Aboriginal community would be appropriate. It could raise awareness among policy makers of the existing barriers to care within the health care system, thus enabling structural and service modifications to meet community needs.

A decision was made collaboratively by HS, ACW, AWP members, the CAN team and supervisors at the AWP meeting to predominantly use critical theory as the overarching theory for this comorbidity study and to look very deeply at the grassroots level using Dadirri and Ganma. There was consensus that this would provide the best opportunity to understand the primary causes of power imbalances linked to colonisation and how these impacted on MH and AOD comorbidity care access. Collectively, the concepts Yarning, Dadirri, Ganma, community development, self-determination, partnership, critical theory and post-colonial theory were brought together to inform and develop an approach to PAR most appropriate for exploring and potentially improving MH and AOD comorbidity services with, and for, Aboriginal peoples. This approach was adopted to enable Aboriginal community members’ full engagement with the research process, integration of their knowledge and approaches, and inbuilt flexibility and sustainability that respected their needs in terms of fulfilling their cultural responsibilities. Importantly, it also had the potential to accelerate the shift towards local Aboriginal community members being recognised as accountable partners, advisors and advocates in research.

## Results

### Constructing a PAR framework

HS adapted Kemmis, McTaggart and Nixon’s [16] reflective framework of communicative action and incorporated Dadirri and Ganma throughout each of four phases, as shown in Fig. 1. Understanding how each element of Dadirri interacted with PAR enabled HS to co-develop a respectful approach with ACW and the AWP. Together they listened to the diverse voices of local Aboriginal people respectfully and democratically [25, 26, 38]. Closely aligned with Freire’s concept that the ‘best way to learn is by doing, and the best way to do is by learning’ [39], the amalgamation of Dadirri and PAR enabled information and knowledge to be shared and interpreted at the same time. As the research process progressed, HS discovered the methodological elements of Dadirri that correlated with the elements of PAR, as illustrated in Fig. 2. Figures 1 and 2 may be useful for researchers working with communities in need. Moreover, Dadirri can be used as a stand-alone methodology.

### Using the methodology

Once developed, the methodology was used to begin conducting the research project, and HS, ACW and the AWP continued to reflect on and refine the methodology in response to interactions and findings within the research project. This interaction closely aligns with Rigney’s [40, 41] position that Aboriginal people’s diverse experience should underpin construction of the methodology – their voices must be heard. The Aboriginal Health Research Ethics Committee’s expectation is that research processes are responsive to need [42].

Repeated cycles of look and listen, think, discuss, collaborate, consult, plan and take action were used throughout the project, as illustrated in Table 1. Concepts of Yarning, Ganma and Dadirri underpinned all interactions, and critical and post-colonial theory helped to shape interpretation and action.

The ethical dimensions were balanced by honouring the mutual research relationship, co-producing findings of the project, developing recommendations with ‘all participants’ (community members, comorbidity and support service staff) while being mindful of Western dominance. HS, ACW and the AWP discussed the need to create a ‘fair play ground’ where all information was acknowledged and respected. Using principles of Yarning, Dadirri and Ganma they discussed between themselves, and with other participants and key stakeholders, the best way forward. Key stakeholders included members of the wider CAN research project, the Aboriginal Health Council of South Australia and the local council.

The final workshop was held in the local area, enabling local Aboriginal participants to be involved in decision making more easily and meaningfully. Support service providers, their managers and other key stakeholders attended. There was an open invitation for anyone interested to be involved in planning the workshop and local Aboriginal people joined the workshop. Each AWP member chaired each workshop table comprising of 10 participants. Two staff from the Aboriginal Health Council of South Australia facilitated the workshop to create a safe space for Aboriginal community members to address power dynamics, and skilfully enable complex conversations toward agreed recommendations. Respectful listening (Dadirri) and self-reflective knowledge sharing (Ganma) underpinned the approach. Small table discussions and note taking (Yarning) enabled all opinions and feedback to be heard. The workshop planning, facilitation and agreed recommendations involved diverse groups and became an integral part of the whole PAR process, reinforcing and incorporating the methodology chosen.

## Discussion

Aboriginal people have been researching and working together, reviewing and improving the health and wellbeing of their communities for thousands of years, and they shared this collaborative approach with HS. Applying PAR and Dadirri methodology using Yarning and Ganma helped HS to understand the concepts theoretically, which assisted her to work alongside ACW and the AWP. This way of working enabled her to learn and practice the skills of utilising these approaches. HS learned how to work respectfully in this collaborative intercultural space whilst also meeting deadlines and budgets. She learned the important skill of facilitating by ‘gently holding’ a group of people together, exhibiting their own freedom and uniqueness as described in Ganma, (not to force anyone into research or for anyone to carry others’ voices). Listening to and valuing everyone’s opinion, and reaching consensus are essential, as informed by Dadirri.

This project was built collaboratively in consultation (Yarning) from the ground up, focused on an issue of great concern to the local Aboriginal community participating in this research; an urgent need for a restructure of MH-AOD services in line with the project’s findings. The project had the potential to inform service improvements and system changes. As such, it was not primarily focused on the needs and priorities of the PhD student; rather, it was focused on the needs and priorities of Aboriginal community members and the challenges facing MH-AOD staff and services. The collaborative decision to use Western critical theory in conjunction with the Aboriginal concepts Yarning, Dadirri and Ganma facilitated development of the strong, trusting partnership between HS, Aunty Coral Wilson (ACW, the Aboriginal Elder/mentor/researcher) and the Aboriginal Working Party (AWP) that was central to the success of co-designing a new methodology and the methods used to investigate MH-AOD comorbidity care. Ongoing, transparent communication, two-way knowledge sharing and decision making helped to build trust between team members, and the team and the wider Aboriginal community and service providers. This process was as important as the findings because it enabled a deeper discussion and identification of the different elements needed to restructure services. While the PAR methodology was to be applied in the academia of her PhD, HS discovered, learnt to understand and practiced the components of Dadirri, with each step cross checked, approved by the AWP members and further confirmed by the local wider Aboriginal community members.

HS found that she needed to develop strategies that enabled her to listen deeply to emotionally distressing information, and then balance responding to Aboriginal participants’ individual issues with the collective needs of diverse Aboriginal participants and stakeholders, and the role of research coordinator. ACW and the AWP assisted her in developing strategies because these are the kind of challenges they face daily in their own work. At a personal level, these deep relationships and deep listening posed a significant challenge for HS. It took many meetings with Aboriginal and non-Aboriginal people before HS felt she had sufficient understanding to develop the authentic, meaningful relationships and collaborative research processes required for this type of research to succeed. Meetings happened at the University, coffee shop, mall, park land, convention centre, health services, community centres and houses. HS recollects interviewing an Aboriginal lady who was camping in the park land. The interview took place while they were walking but they both engaged in deep conversation. HS was also invited to Aboriginal church. These meetings not only facilitated gathering information to address people’s everyday experiences of MH and AOD issues for the improvement of comorbidity services but they were part of the AWP and local Aboriginal people guiding HS in her PhD journey. Working and writing in a Western way was as new for HS as learning to work with the Aboriginal methodologies of Dadirri and Ganma, using Yarning. Along her journey, HS came to learn the Aboriginal way and Western way, and to meld both together in the PAR-Dadirri-Ganma research model. One remarkable success of this project was that one of the Aboriginal women went on to undertake higher studies at university.

One ethical concern that is often raised in relation to collaborative research involving Aboriginal community members is remuneration and recognition [43]. Adequate funds are crucial for truly collaborative approaches with Indigenous governance and decision making to be acceptable and effective. In this research project, the university employed ACW as a co-researcher and mentor, and the majority of the AWP were employed in MH and AOD or support roles. They attended meetings and workshops as part of their working hours, with the support of their team leaders and managers. Interviews and focus groups with community members were held at a time and location most suitable for participants (to reduce inconvenience). Although participants were not financially remunerated (a decision supported by the wider CAN project and the Aboriginal Health Research Ethics Committee at the time), refreshments and transport assistance were provided, particularly at the longer final workshop in which community members and local service providers participated. The local council provided the workshop venue free of charge, transport assistance was arranged through a variety of means, and catering was provided from the project budget. Feedback from community members was that they attended because they saw HS’s genuine concern for, and engagement with, their shared experiences of MH-AOD service provision, and they saw the potential for this PAR-Dadirri-Ganma project to make a change. They also discussed how they had been involved in repeated studies that paid ‘lip service’ to their concerns, but no action to improve access or services had been taken. In this case, the study’s recommendations were taken for consideration by the South Australian Parliament.

Another concern often raised is whether each participant’s voice is heard equally. The methodology and methods used in this project addressed this in several ways. While ACW was an Elder, mentor and co-researcher, she firmly believed in the importance of a range of voices being heard. The AWP used collective decision-making processes that respected the Elder’s leadership but also ensured that all voices were respected and a range of opinions were incorporated into each decision. The concepts and practices of Dadirri and Gama were embedded into the way the research team members interacted among themselves, as well as how the research incorporated the viewpoints of wider community members and MH-AOD and support service staff in interviews, focus groups and the final workshop. Dadirri and Ganma underpinned all stages of the research, beginning with planning and data collection through to analysis and writing recommendations for action.

This process reminded HS, as a non-Aboriginal researcher, to avoid making assumptions and unilateral decisions about what she thought might be most appropriate for the AWP, other participants and the wider Aboriginal community in terms of the research process and recommendations for improvements in comorbidity care.

This mindful approach arose from the reflexivity built into the research process through its foundations of Yarning, Dadirri and Ganma; constant communication with, and gentle support from ACW and members of the AWP. This enabled HS to overcome her initial feelings of being lost, and to acknowledge and confront her “vulnerabilities and mistakes” [44]. She was determined to learn from her Aboriginal co-researchers. As HS took each step along the research journey, and she developed stronger working and personal relationships with her research partners, her position gradually changed from her perception of herself as an outsider, novice researcher to an accepted and valued member of the research team. However, becoming comfortable did not reduce her awareness that she needed to attend each meeting with an open mind, to communicate honestly and considerately, and focus on the way she shared information and the way she listened to others. HS appreciated the support and guidance of ACW, and learnt to “go with the flow”, adapting as necessary according to the Aboriginal researchers’ family, personal and cultural commitments, taking the time to “be human” instead of focusing solely on achieving the unrealistic deadlines that so often drive Western research (and must be met to satisfy funders). The process taught her that there was no place in PAR-Dadirri-Ganma for working alone. She came to learn from ACW and the Aboriginal researchers, to understand and to practice what Wilson called ‘relational accountability’ in his Indigenous research paradigm [19]. All voices deserved and received equal respect, and the collaborative contributions of many people as opposed to only one main researcher made for more rigorous research from the beginning until the end, and multiple perspectives on how to improve MH-AOD services for Aboriginal communities.

### Research limitations

The more recent development of methodologies and increasing use of Indigenous methodologies and methods such as Yarning, Dadirri and Ganma offers new approaches and understanding for Aboriginal health research, and provides a clearer positioning of Indigenous and non-Indigenous researchers. Future research projects should endeavour to include an increased budget to cover payment for community member participation (sitting fees, transport and catering costs). With the increase in the number of Aboriginal and Torres Strait Islander researchers and higher degree students, there has been a much-needed prioritisation of Indigenous people undertaking Indigenous research. However, the extent and breadth of health care challenges and current ethical agreements are such that within Australasia, non-Indigenous researchers will still be actively involved, at times as PhD candidates. Truly collaborative approaches to Indigenous governance and decision making that represent diverse Aboriginal viewpoints and ages are crucial. However, adequate and sustainable funding is essential for these to be acceptable and effective.

## Conclusions

This paper describes the process of co-design undertaken by an international PhD student working in deep collaboration with Aboriginal mentors and an Aboriginal Working Party in a MH-AOD comorbidity needs study. Unlike traditional hegemonic research, this collaborative PAR was underpinned by Indigenous concepts of Yarning (talking together), Dadirri (deep listening) and Ganma (knowledge sharing) in conjunction with critical theory. This approach enabled and reflected a mutual and trustworthy partnership and reciprocal relationship, resulting in a research methodology that was fit for purpose; it encouraged diverse participants to speak out, be heard and actively co-design recommendations for changes in care provision. It is hoped that this example of genuine research collaboration among the very people impacted by the scourge of MH-AOD comorbidity (largely resulting from the effects of colonisation and compounded by inadequate service provision), service providers and an international PhD student, will provoke serious questioning among the research community about how to learn to elicit the deepest knowledge, truest partnerships and most workable solutions to problems experienced by diverse communities world wide. Further, HS aspires to apply Dadirri as an Aboriginal methodology equalling PAR in future qualitative research.

## Data Availability

Yes.

## Footnotes

2 Aunty Coral Wilson passed away in 2017. She had read a first draft and given the main author HS permission to complete and submit this article for publication with approval from all others involved in the research. Aunty Coral was very strong in her directive to HS that her name be used because she stressed how important this research was in trying to achieve better health services for her community, and to develop a better way of undertaking Aboriginal research. Her family also gave permission.
